# Vitamin B12 combined with methylene blue alleviates experimental septic shock and is associated with modulation of KCNMB1 and the cGMP-PRKG pathway

**DOI:** 10.1371/journal.pone.0355117

**Published:** 2026-08-06

**Authors:** Keji Shan, Jinling Zhao, Kun Tang, Yuying Lei, Fukang Mao

**Affiliations:** Department of Intensive Care Unit, The First Affiliated Hospital of Kunming Medical University, Kunming, Yunnan, China; University of Westminster - Regent Street Campus: University of Westminster, UNITED KINGDOM OF GREAT BRITAIN AND NORTHERN IRELAND

## Abstract

**Background:**

Septic shock is characterized by severe circulatory failure, microvascular dysfunction, and metabolic derangement. Methylene blue (MB) and vitamin B12 (VB12) have each shown potential benefit in vasodilatory shock, but the biological effects of their combined use remain unclear.

**Methods:**

A rat model of LPS-induced septic shock was used to evaluate the effects of combined MB and VB12 pretreatment. Transcriptomic analysis of carotid artery tissue was performed to identify treatment-responsive genes and pathways. Expression of KCNMB1, PRKG1, and PRKG2 was assessed by immunohistochemistry, western blotting, and RT-qPCR. Hemodynamic parameters, circulating injury markers, inflammatory cytokines, oxidative stress indices, and histological changes in major organs were also evaluated.

**Results:**

Combined MB and VB12 treatment was associated with lower expression of KCNMB1, PRKG1, and PRKG2 and reduced cGMP levels compared with the model group. The combined treatment also partially improved hemodynamic abnormalities, reduced NO, H2S, and lactate levels, and attenuated inflammatory and oxidative stress responses. Histological injury in the lung, liver, kidney, and carotid artery was also alleviated.

**Conclusion:**

In this experimental model, combined MB and VB12 pretreatment attenuated multiple features of septic shock and was accompanied by modulation of KCNMB1 and cGMP-PRKG pathway-related markers. These findings support a potential protective role of the combined regimen, although the causal mechanistic relationship requires further investigation.

## Introduction

Sepsis is defined as life-threatening organ dysfunction caused by a dysregulated host response to infection, whereas septic shock is a severe subset characterized by circulatory, cellular, and metabolic abnormalities with increased mortality risk. According to the latest Surviving Sepsis Campaign Guidelines, early recognition, infection control, and hemodynamic resuscitation remain central to the management of sepsis and septic shock [1]. Common underlying mechanisms involve systemic inflammatory responses triggered by infection, widespread microvascular dysfunction, tissue hypoperfusion, and subsequent metabolic imbalance [2]. Globally, sepsis is responsible for approximately 11 million deaths annually, with related healthcare expenditures in the United States alone exceeding $38 billion. Improved diagnostic and therapeutic strategies have the potential to shorten ICU stays and reduce healthcare resource utilization [3].

In recent years, methylene blue (MB) has emerged as a promising non-catecholamine vasopressor with clinical potential in the management of septic shock. MB counteracts excessive nitric oxide (NO) production, thereby attenuating pathological vasodilation and improving tissue perfusion and organ function [4]. Moreover, MB possesses additional properties, including antioxidant activity, mitochondrial protection, anti-inflammatory effects, and enhancement of microcirculatory flow [5,6]. Unlike non-selective nitric oxide synthase inhibitors (such as L-NMMA and L-NNA), MB specifically inhibits soluble guanylate cyclase (sGC), which catalyzes cyclic guanosine monophosphate (cGMP) synthesis in response to NO stimulation. Elevated cGMP levels lead to vasodilation and suppression of vascular smooth muscle proliferation [7,8]. Importantly, one of the most clinically relevant effects of MB is its potential vasopressor-sparing effect. Recent studies and systematic reviews have suggested that MB may improve hemodynamic stability, reduce vasopressor requirements, shorten the time to vasopressor discontinuation, and improve short-term outcomes in patients with vasodilatory shock or septic shock [9–11].

Vitamin B12 (VB12), a water-soluble vitamin, exhibits a wide range of pharmacological effects, including the promotion of erythropoiesis, prevention of anemia, neuroprotection, anti-inflammatory actions, and reduction of H2S and NO bioavailability [12,13]. Recent clinical trials have demonstrated that high-dose intravenous hydroxocobalamin significantly reduces vasopressor requirements and circulating H_2_S levels compared to placebo in patients with septic shock, without severe adverse events, thereby supporting its therapeutic potential [14]. Based on these findings, we hypothesized that the combined use of MB and VB12 may provide complementary effects in septic shock. However, available clinical evidence mainly supports the separate use of either MB or hydroxocobalamin, whereas direct clinical evidence for their combined administration remains limited. Therefore, further experimental studies are needed to evaluate the biological effects and potential mechanisms of combined MB and VB12 administration.

Building on these clinical observations, we investigated the biological effects of combined MB and VB12 pretreatment in a rat model of LPS-induced septic shock. Transcriptomic analysis of carotid artery tissue identified KCNMB1, PRKG1, and PRKG2 as treatment-responsive genes. We then assessed whether modulation of these genes was accompanied by improvements in hemodynamic parameters, tissue injury, systemic inflammation, and oxidative stress. Our study was designed to explore a potential mechanism associated with the combined treatment rather than to establish a definitive causal signaling axis.

## 2. Materials and Methods

### 2.1. RNA-seq

RNA was extracted and subjected to quality control before library preparation. mRNA was enriched using oligo(dT) magnetic beads, fragmented, and reverse-transcribed to synthesize double-stranded cDNA. After end repair, A-tailing, and adapter ligation, libraries were size-selected and PCR-amplified. Library quality was assessed using Qubit 3.0, Qsep400, and qPCR. Qualified libraries were sequenced on an Illumina platform (PE150). Raw reads were filtered to obtain clean data, which were aligned to the reference genome. Downstream analyses, including differential expression, functional annotation, enrichment, and PCA, were performed using the BMKCloud platform (www.biocloud.net).

### 2.2. Animal

Male Sprague-Dawley (SD) rats (n = 30; body weight: 200 ± 20 g) were purchased from SPF (Beijing) Biotechnology Co., Ltd. and acclimated for one week under standard laboratory conditions (temperature: 22 ± 2 °C; 12 h light/dark cycle) with free access to food and water. First, rats were administered VB12 (1.5 mg/kg, i.p.) and/or MB (4 mg/kg, i.p.) via intraperitoneal injection for three consecutive weeks. Following a three-week treatment with VB12 and/or MB, the septic shock model was established on the final day. Immediately after the last administration of the treatment, rats received an injection of lipopolysaccharide (LPS, 10 mg/kg) via the external jugular vein. The model was considered successfully established when the mean arterial pressure (MAP) decreased by 20%−30% from baseline or dropped to ＜ 65 mmHg and was sustained for more than 10 minutes.

Cardiac function and hemodynamic parameters were assessed at 2, 4, and 6 hours after the successful establishment of the model. A pressure transducer connected to an eight-channel physiological recorder was utilized. The right carotid artery was cannulated to monitor systolic blood pressure (SBP), diastolic blood pressure (DBP), and MAP. A Millar catheter was introduced via the right carotid artery into the left ventricle to determine cardiac output (CO). Additionally, peripheral vascular resistance (PVR) was calculated at each time point. To avoid potential interference from catheterization-related vascular injury, the catheterized right carotid artery was not used for subsequent histological, transcriptomic, or molecular analyses. The contralateral left carotid artery, which was not used for catheterization, was collected for downstream analyses.

At 24 hours post-modeling, the contralateral left carotid artery, liver, kidney, and lung tissues were collected, divided into two portions, and either fixed in 4% paraformaldehyde for histological examination or snap-frozen in liquid nitrogen for subsequent molecular analyses.

### 2.3. Grouping and Modeling

Rats were randomly assigned to five groups (n = 6 per group): Sham, Model, VB12, MB, and VB12 + MB. Animals in the VB12, MB, and VB12 + MB groups received daily intraperitoneal injections of VB12 (1.5 mg/kg/d), MB (4 mg/kg/d), or a combination of both for three consecutive weeks before LPS challenge; the other groups were given an equivalent volume of normal saline. Except for the Sham group, all rats underwent the septic shock procedure. The Sham group received the same surgical interventions but only received saline without active agents.

The dose of MB was selected because 4 mg/kg has been used in experimental studies evaluating the anti-inflammatory, vascular, and microcirculatory effects of MB, and recent clinical studies have also evaluated MB bolus doses within the 1–4 mg/kg range in septic shock. The dose of VB12 was selected based on previous rodent studies in which 1.5 mg/kg/day cyanocobalamin or methylcobalamin produced protective effects against tissue injury, inflammation, or oxidative stress. These doses were therefore chosen as biologically active exploratory doses for evaluating the combined effects of MB and VB12, rather than as optimized doses derived from a formal dose-response analysis [15–17].

For modeling, after three weeks of pre-treatment, rats were fasted for 12 hours with free access to water. Animals were anesthetized with 3% pentobarbital sodium (30 mg/kg, intraperitoneally). Then, lipopolysaccharide (LPS, 10 mg/kg) was administered via external jugular vein injection. A pressure transducer connected to an eight-channel physiological recorder was used to continuously monitor systolic blood pressure, diastolic blood pressure, and mean arterial pressure (MAP) via catheterization of the right carotid artery. A Millar catheter was introduced via the right carotid artery into the left ventricle to determine cardiac output (CO). Peripheral vascular resistance (PVR) was calculated as MAP divided by CO at each time point. Upon confirmation of septic shock, resuscitation was initiated with lactated Ringer’s solution (30 ml/kg) infused over 30 minutes and norepinephrine at 0.25 μg/kg/min for 2 hours. Blood samples were collected from the tail vein every 2 hours and at 24 hours post-modeling. The contralateral left carotid artery, liver, kidney, and lung tissues were then harvested for further analyses.

### 2.4. Animal welfare, monitoring, and mortality

This study used an acute LPS-induced septic shock model with a fixed 24-h observation period. Humane endpoint criteria were predefined, including persistent unresponsiveness to external stimuli, severe respiratory distress, inability to maintain sternal recumbency, or other signs of severe and irreversible distress as judged by trained personnel. All animal procedures, including animal monitoring procedures and endpoint criteria, were reviewed and approved by the Ethical Review System for Laboratory Animal Welfare of Wuhan Myhalic Biotechnology Co., Ltd. (Approval No. HLK-20250210–001). All procedures were carried out in accordance with institutional guidelines for the care and use of laboratory animals.

Animals were monitored at least twice daily during the 3-week pretreatment period. On the day of modeling and throughout the acute post-LPS phase, animals were closely observed during the procedure and monitored at least every 2 h until the planned 24-h endpoint, in conjunction with blood sampling and hemodynamic assessment. General condition, activity, posture, responsiveness, and respiratory status were recorded throughout the experiment.

For anesthesia, all invasive procedures, including vascular catheterization, LPS administration, hemodynamic monitoring, and blood sampling, were performed under pentobarbital sodium anesthesia. Animals were anesthetized with 3% pentobarbital sodium at 30 mg/kg by intraperitoneal injection before surgical manipulation. Adequate depth of anesthesia was confirmed by the absence of pedal withdrawal and corneal reflexes before invasive procedures were initiated.

A total of 30 rats were used in this study (n = 6 per group). No animals died before the planned endpoint, and no animals required early euthanasia based on the predefined humane endpoint criteria. At the planned 24-h experimental endpoint, animals were euthanized under deep pentobarbital sodium anesthesia before tissue collection. Death was confirmed by cessation of respiration and heartbeat before tissue harvesting.

To minimize pain and distress, all invasive procedures were performed under anesthesia, animals were monitored regularly during the pretreatment and acute post-LPS periods, and supportive treatment, including lactated Ringer’s solution and norepinephrine, was administered after shock induction according to the study protocol. Animals were housed under standard laboratory conditions with free access to food and water except for the pre-model fasting period. All efforts were made to minimize suffering and reduce the number of animals used. Animal procedures and monitoring were carried out by trained personnel experienced in rodent handling, anesthesia, peri-procedural monitoring, and endpoint assessment.

### 2.5. RT-qPCR

Total RNA was extracted from snap‑frozen rat carotid artery tissues using TRIzol reagent (TRANS, ER501−01) according to the manufacturer’s instructions, and RNA purity and concentration were assessed by A260/A280 ratio on a NanoDrop spectrophotometer. First‑strand cDNA was synthesized from 1 µg of total RNA using the PrimeScript™ RT reagent kit (TRANS, AU341) under the following conditions: 37 °C for 15 min and 85 °C for 5 s. Quantitative PCR was performed on a CFX96 Real‑Time PCR System (Bio‑Rad) using SYBR® Premix Ex Taq™ II (Takara) with gene‑specific primers (forward and reverse, 200 nM each) in a 20 µL reaction volume. The thermal cycling protocol comprised an initial denaturation at 95 °C for 30 s, followed by 40 cycles of 95 °C for 5 s and 60 °C for 30 s. Relative expression levels were calculated using the 2^–ΔΔCt^ method with GAPDH as the internal control. The primer sequences used for RT-qPCR are listed in Table 1.

**Table 1:** Primer sequences used for RT-qPCR.

Gene	Primer	Sequence (5′–3′)
KCNMB1	KCNMB1-F	GAAGACACTCGGGATCAAAAC
KCNMB1	KCNMB1-R	AAGAAGGAGAAGAGGAGGATTT
PRKG1	PRKG1-F	CGAGAAGACTCACCAAGTGAAGA
PRKG1	PRKG1-R	TAGTCCAGAGTTCTCCACCTAGG
PRKG2	PRKG2-F	GAGGACGCCGAGACGC
PRKG2	PRKG2-R	ATGTTGCTCAGGAACCAGGC
GAPDH	GAPDH-F	TGCCACTCAGAAGACTGTGG
GAPDH	GAPDH-R	TTCAGCTCTGGGATGACCTT

### 2.6. Western blot

Protein was extracted from frozen carotid artery samples by homogenization in RIPA buffer containing protease and phosphatase inhibitors, and lysates were centrifuged at 12,000 × g for 15 min at 4 °C. The supernatant was collected and protein concentration determined by BCA assay (Pierce). Equal amounts (30 µg) of protein were separated on 10% SDS–PAGE gels and transferred onto PVDF membranes (Millipore) at 100 V for 90 min. Membranes were blocked with 5% non‑fat milk in TBS‑T (Tris‑buffered saline with 0.1% Tween‑20) for 1 h at room temperature, then incubated overnight at 4 °C with primary antibodies KCNMB1 (Proteintech, 12933–1-AP, 1:1,000), PRKG1 (Proteintech, 21646–1-AP, 1:1,000), PRKG2 (Proteintech, 55138–1-AP, 1:1,000), β‑actin (Abcam, ab8226, 1:5,000). After washing, membranes were incubated with HRP‑conjugated secondary antibodies (Abcam, ab6721, 1:5,000) for 1.5 h at room temperature. Protein bands were visualized using ECL substrate (Thermo Fisher) and quantified by densitometry with ImageJ software, normalizing to β‑actin as a loading control.

### 2.7. ELISA

Frozen carotid artery tissues were homogenized in 0.1 M HCl, centrifuged at 10,000 × g for 10 min at 4 °C, and supernatants collected for analysis. A competitive cGMP ELISA kit (Abcam, ab323638) was used according to the manufacturer’s protocol. Standards and samples (50 µL) were added to cGMP-coated wells and incubated with cGMP peroxidase conjugate for 2 h at room temperature. Wells were washed 5 times with wash buffer before addition of TMB substrate, and color development was stopped after 15 min with 2 M sulfuric acid. Absorbance was measured at 450 nm, and cGMP concentrations were calculated from a standard curve using four-parameter logistic regression. cGMP levels were normalized to the total protein concentration of each tissue homogenate.

### 2.8. Measurement of serum biochemical, inflammatory, and oxidative stress markers

Serum samples were collected at the indicated time points and centrifuged at 3,000 × g for 10 min at 4 °C. The supernatants were stored at −80 °C until analysis. Serum Syndecan-1, IL-1, and IL-6 levels were measured using commercially available ELISA kits according to the manufacturers’ instructions. Serum albumin and lactate levels were measured using biochemical assay kits. NO and H2S levels were determined using colorimetric assay kits. Oxidative stress-related indicators, including ROS, SOD, GSH-Px, and CAT, were measured using corresponding assay kits according to the manufacturers’ protocols. Absorbance or fluorescence was measured using a microplate reader, and concentrations or activities were calculated according to standard curves or kit instructions.

### 2.9. Immunohistochemistry

Paraffin‑embedded carotid artery sections (4 µm) were deparaffinized in xylene (3 × 5 min) and rehydrated through graded ethanol to distilled water. Antigen retrieval was performed by heating sections in 10 mM citrate buffer (pH 6.0) at 95 °C for 20 min, followed by cooling to room temperature. Endogenous peroxidase activity was quenched with 3% H_2_O_2_ in methanol for 10 min. Non‑specific binding was blocked with 5% bovine serum albumin in TBS‑T for 1 h at room temperature. Sections were incubated overnight at 4 °C with primary antibodies against KCNMB1, PRKG1, PRKG2, TGFBR1, and PDGFB (1:200). After washing, sections were incubated with HRP‑conjugated secondary antibody (1:500) for 1 h at room temperature and developed with DAB substrate (Thermo Scientific, 34002). Stained sections were counterstained with hematoxylin, dehydrated, cleared, and mounted for light microscopic (Olympus BX63).

### 2.10. HE staining

Paraffin-embedded tissue sections from the lung, liver, kidney, and carotid artery were cut at 4 µm, deparaffinized in xylene, and rehydrated through a graded ethanol series to distilled water; nuclei were stained with Mayer’s hematoxylin (Beyotime, Y269827) for 5 min, rinsed in running tap water for 2 min, and differentiated in 0.5% acid alcohol for 5 s followed by another tap‑water rinse. Sections were then counterstained with 1% eosin Y (Beyotime, Y263074) for 2 min, dehydrated through ascending ethanol, cleared in xylene (2 × 5 min), and mounted with a synthetic resin. Stained slides were examined under a light microscope (NanoZoomer 2.0-HT) to assess morphological changes such as intimal thickening and inflammatory cell infiltration.

### 2.11. Statistical analysis

Data are presented as mean ± standard deviation (SD). Statistical analyses were performed using GraphPad Prism 8.0 (GraphPad Software, USA). The normality of data distribution was assessed using the Shapiro-Wilk test. For normally distributed single-time-point data, comparisons among multiple groups were performed using one-way analysis of variance (ANOVA) followed by Tukey’s post hoc test. For data that did not satisfy the assumption of normality, non-parametric tests were used instead of data transformation; specifically, the Kruskal-Wallis test followed by Dunn’s multiple-comparison test was applied for comparisons among multiple groups. For measurements obtained across multiple time points, two-way repeated-measures ANOVA was used when model assumptions were met, with treatment group and time as the main factors, followed by appropriate multiple-comparison testing. For repeated-measures data with more than two time points, the assumption of sphericity was assessed using Mauchly’s test. When the sphericity assumption was violated, the Greenhouse-Geisser correction was applied to adjust the degrees of freedom. If the normality assumption was not met for time-course variables, group comparisons were further assessed at individual time points using non-parametric tests. A two-sided p value < 0.05 was considered statistically significant. The number of biological replicates for each assay is indicated in the figure legends. Technical replicates, when performed, were averaged before statistical analysis.

## 3. Results

### 3.1. Transcriptome analysis

**Fig 1:**
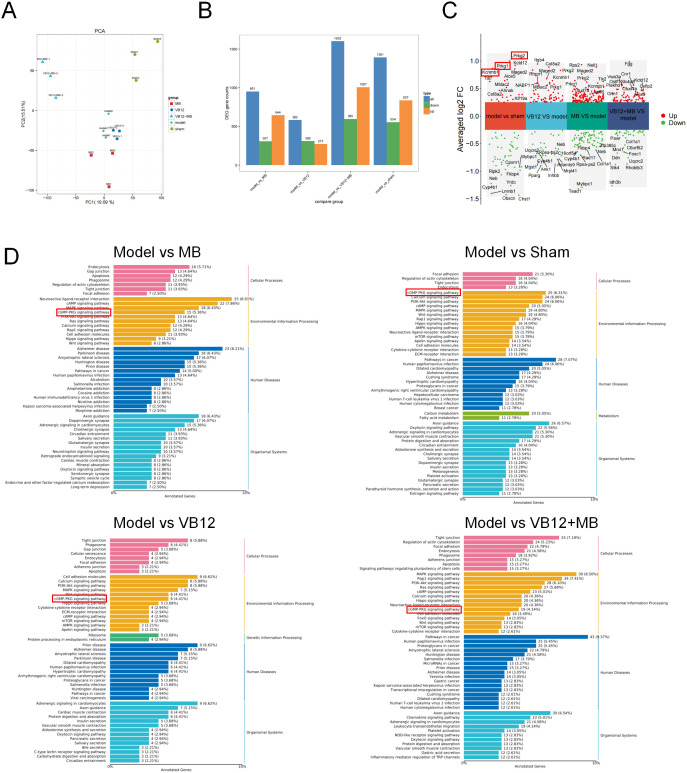
Transcriptomic analysis of carotid artery tissues after VB12 and MB administration. (**A**) PCA plot of transcriptomes. (**B**) Count of different genes, model vs VB12, MB, VB12 + MB group. (**C**) Volcano plot of differentially expressed genes (DEGs). (**D**) KEGG enrichment analysis of UP genes.

To investigate the effects of combined VB12 and MB administration on septic shock in rats, we performed transcriptomic analysis of carotid artery tissues. Principal component analysis (PCA) revealed distinct clustering among the different groups, indicating significant differences in gene expression profiles (Fig 1A). Differential gene expression analysis showed substantial numbers of upregulated and downregulated genes when comparing the model group with both the treatment and sham groups. The largest number of differentially expressed genes (DEGs) was observed between the model group and the combination treatment group (Fig 1B).

We further examined specific genes of interest and identified KCNMB1 and PRKG as significantly upregulated in the model group compared with the sham group (Fig 1C). Pathway enrichment analysis of the upregulated genes showed enrichment of the cGMP-PRKG signaling pathway across group comparisons (Fig 1D).

### 3.2. Combined VB12 and MB treatment modulates KCNMB1 and cGMP/PRKG pathway-related markers in carotid artery tissue

**Fig 2:**
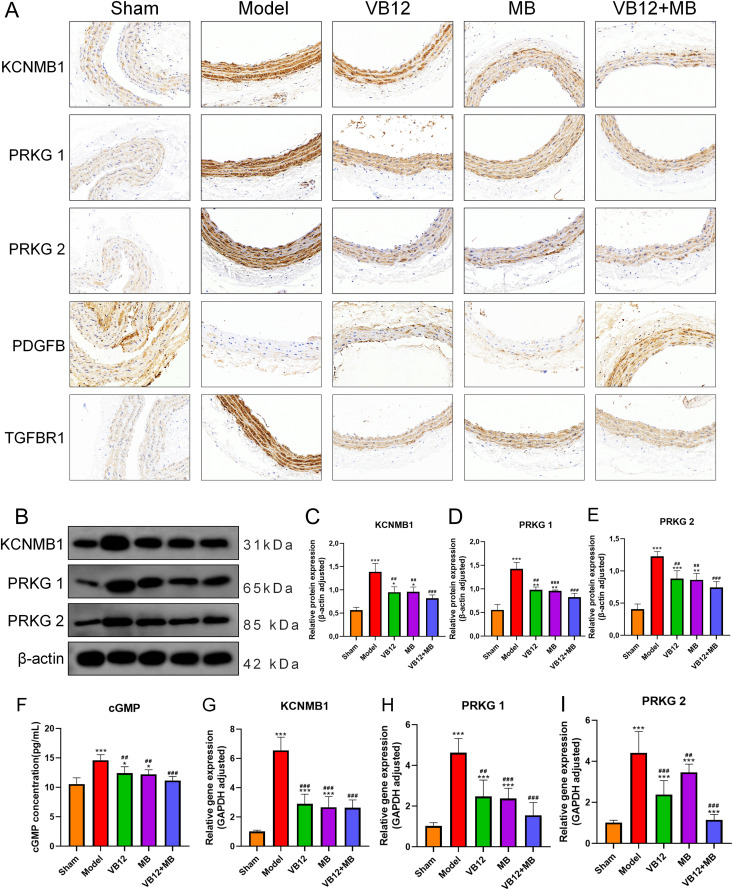
Combined vitamin B12 and methylene blue treatment modulates KCNMB1 and cGMP-PRKG pathway-related markers in carotid artery tissue. (**A**) Immunohistochemical staining of KCNMB1, PRKG1, PRKG2, PDGFB, and TGFBR1 in rat carotid arteries (n = 6/group). (**B**) Representative western blot bands for KCNMB1, PRKG1, and PRKG2, and β-actin. (**C-E**) Quantification of relative protein levels of KCNMB1, PRKG1, and PRKG2 (n = 3/group). (**F**) cGMP concentration measured by ELISA (n = 6/group). (**G-I**) Relative mRNA expression levels of KCNMB1, PRKG1, and PRKG2 determined by RT-qPCR. Data are presented as mean ± SD.

To evaluate the expression of KCNMB1, PRKG1, PRKG2, PDGFB, and TGFBR1 in carotid artery tissues, immunohistochemistry was performed. Compared with the sham group, the model group showed stronger staining for KCNMB1, PRKG1, PRKG2, and TGFBR1, whereas PDGFB staining was reduced. Treatment with VB12 or MB altered these staining patterns, and the combined treatment group showed lower KCNMB1, PRKG1, PRKG2, and TGFBR1 staining and higher PDGFB staining than the model group (Fig 2A).

Consistent with the IHC findings, western blotting and RT-qPCR showed that KCNMB1, PRKG1, and PRKG2 protein and mRNA levels were increased in the model group compared with the sham group. VB12 or MB treatment reduced their expression levels, with the combined treatment group showing the lowest levels among the LPS-challenged groups (**Fig 2B–I**).

### 3.3. Combined VB12 and MB treatment partially improves hemodynamic abnormalities and circulating injury markers in septic shock rats

**Fig 3:**
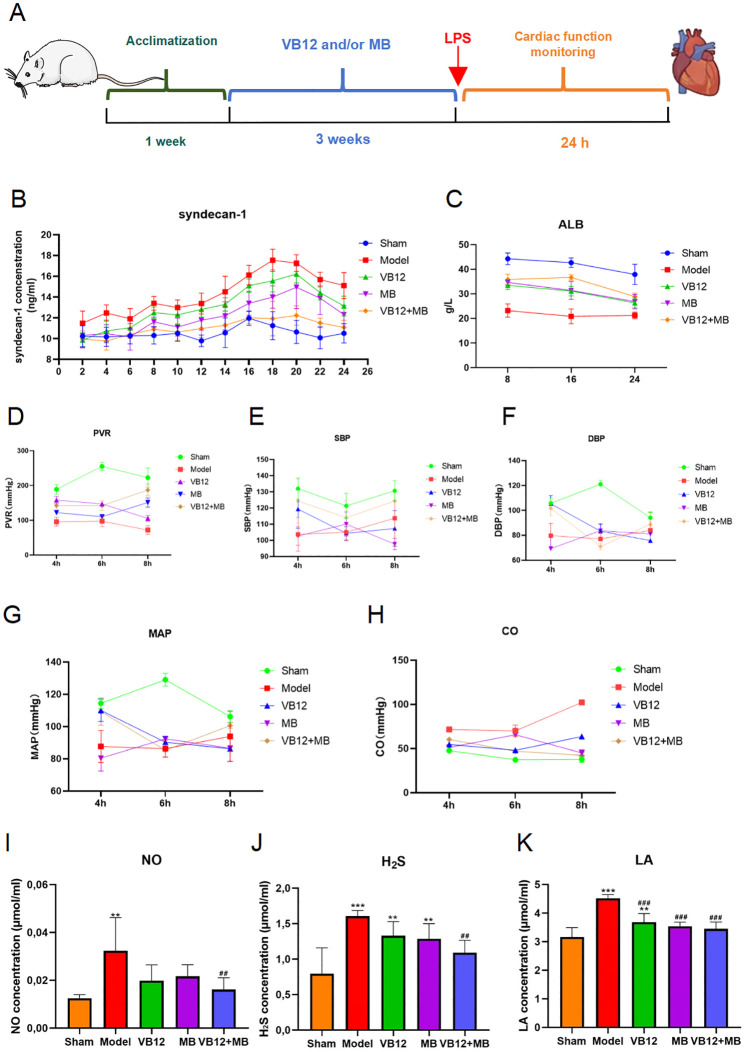
Vitamin B12 combined with methylene blue partially improves hemodynamic abnormalities in septic shock rats. (**A**) Experimental design. (**B**) Dynamic serum Syndecan-1 levels after LPS challenge (n = 6/group). (**C**) Serum albumin levels at 8, 16, and 24 h (n = 4/group). (**D-H**) Hemodynamic parameters, including PVR, SBP, DBP, MAP, and CO (n = 3/group). (**I-K)** Serum NO, H2S, and lactate levels (n = 6/group). Data are presented as mean ± SD. Statistical significance is indicated as follows: *p < 0.05, **p < 0.01, ***p < 0.001 compared with sham group; ^##^*p* < 0.01, ^###^*p* < 0.001 compared with model group.

Fig 3A shows the experimental timeline, including acclimatization, three weeks of VB12 and/or MB administration, LPS challenge, and the 24-h observation period. Serum Syndecan-1 levels increased after LPS challenge, peaked at 18 h, and then gradually decreased. The model group showed the highest Syndecan-1 levels across the observation period. VB12 and MB monotherapy groups showed lower Syndecan-1 levels than the model group, and the combined treatment group showed the lowest levels among the LPS-challenged groups (Fig 3B).

Serum albumin levels decreased over time after LPS challenge in the model group. Compared with the model group, VB12, MB, and VB12 + MB groups showed higher albumin levels at the indicated time points, with a more evident difference in the combined treatment group at 16 h (Fig 3C).

Hemodynamic measurements showed that PVR was lower in the model group than in the sham group after LPS challenge. VB12, MB, and VB12 + MB groups showed higher PVR values than the model group at the indicated time points, with a more evident difference in the combined treatment group at later time points (Fig 3D). SBP, DBP, and MAP were also lower in the model group than in the sham group. MB and VB12 + MB groups showed partial increases in these parameters, whereas the changes in the VB12 group were less pronounced (**Fig 3E–G**). CO showed variable changes across groups and time points, without a consistent advantage in the combined treatment group (Fig 3H).

Serum NO, H2S, and lactate levels were higher in the model group than in the sham group. VB12, MB, and VB12 + MB treatment groups showed lower levels of these markers than the model group, with the combined treatment group generally showing the greatest reduction (**Fig 3I–K**).

### 3.4. Combined VB12 and MB treatment alleviates tissue injury in septic shock rats

**Fig 4:**
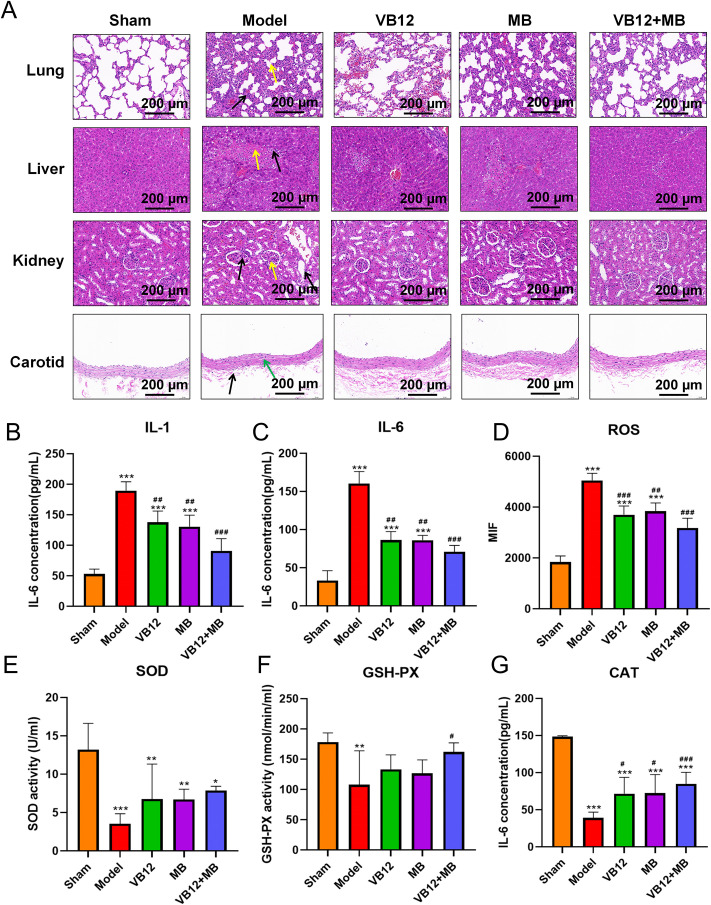
Vitamin B12 combined with methylene blue alleviates tissue injury in septic shock rats. (**A**) HE staining showing histological injury in the lungs, liver, kidneys, and carotid arteries. (**B, C**) Serum levels of inflammatory cytokines IL-1 and IL-6. (**D-G**) Serum levels of oxidative stress-related markers, including ROS, SOD, GSH-Px, and CAT. Data are presented as mean ± SD. Statistical significance is indicated as follows: *p < 0.05, **p < 0.01, ***p < 0.001 vs. Sham group; ##p < 0.01, ###p < 0.001 vs. Model group.

To assess the protective effects of VB12 and MB on target organs, lung, liver, kidney, and carotid artery tissues were harvested for HE staining. In the model group, the lungs exhibited extensive alveolar collapse and shrinkage, marked thickening of alveolar walls, and significant consolidation (yellow arrows). Widespread inflammatory cell infiltration was also observed (black arrows). Both VB12 and MB alleviated alveolar damage and parenchymal changes, while the combined treatment group showed less alveolar collapse, wall thickening, and inflammatory infiltration. Hemorrhagic areas were observed in the VB12 monotherapy group. In liver tissues from the model group, overall structure was severely disrupted with disorganized cellular arrangement and extensive hepatocellular necrosis characterized by karyopyknosis, fragmentation, and dissolution (yellow arrows). Prominent inflammatory infiltration was also evident (black arrows). Treatment with either VB12 or MB partially improved these histopathological changes; however, focal hemorrhage remained observable, whereas hemorrhagic changes were less evident in the combined treatment group. In kidney tissues, the model group showed tubular epithelial injury, tubular dilation, and inflammatory cell infiltration. These renal histopathological changes were attenuated after VB12 or MB treatment and were less pronounced in the combined treatment group. In carotid artery sections, the model group showed endothelial cell deposits (black arrows) and mild inflammatory infiltration (green arrows). VB12 or MB alone reduced these pathological changes (Fig 4A).

To further evaluate systemic effects, levels of circulating inflammatory cytokines and oxidative stress markers were measured. IL-1, IL-6, and ROS were significantly elevated in septic shock rats, whereas both VB12 and MB substantially reduced systemic inflammation and oxidative stress. SOD, GSH-Px, and CAT levels were lower in the model group than in the sham group, whereas VB12, MB, and VB12 + MB treatment groups showed increased levels of these antioxidant enzymes compared with the model group (**Fig 4B-G**).

## Discussion

Septic shock is characterized by infection-induced circulatory, cellular, and metabolic dysfunction and is commonly associated with hypotension, tissue hypoperfusion, organ injury, and metabolic derangement. Its pathogenesis involves systemic inflammatory activation, cardiovascular dysfunction, microcirculatory disturbance, endothelial barrier injury, and coagulation abnormalities [18]. Although current management relies mainly on infection control, hemodynamic support, and organ function maintenance, adjunctive strategies targeting vasoplegia, endothelial dysfunction, and metabolic stress remain clinically relevant. In the present experimental study, combined VB12 and MB pretreatment was associated with attenuation of hemodynamic abnormalities, reduced circulating injury and inflammatory markers, alleviation of multi-organ and carotid artery injury, and decreased expression of KCNMB1, PRKG1, PRKG2, and cGMP-related signaling markers in carotid artery tissue.

A recent systematic review and meta-analysis demonstrated that MB may reduce short-term mortality and vasopressor requirements during septic shock without increasing adverse events, supporting its potential efficacy and safety as an adjunctive vasoactive agent [4]. Similarly, VB12, particularly in the form of hydroxocobalamin, has been reported to reduce vasopressor requirements and H2S levels in patients with septic shock, without severe adverse events [14]. However, it should be emphasized that MB or hydroxocobalamin is generally administered after the onset of septic shock in clinical practice, whereas the present study used a three-week pretreatment design before LPS challenge. This design was not intended to directly reproduce clinical treatment but to serve as a mechanistic and prophylactic experimental model for evaluating whether repeated exposure to VB12 and MB could modify vascular responses, inflammatory activation, oxidative stress, and subsequent shock-related injury. The three-week duration was empirically selected as an intermediate pretreatment period to allow relatively stable biological effects after repeated exposure while avoiding excessive long-term dosing burden in rats. Because different pretreatment durations and post-insult therapeutic administration were not evaluated, the present findings cannot determine whether three weeks represents the optimal duration or be directly extrapolated to clinical therapeutic efficacy.

The selected doses should also be interpreted as exploratory. Although MB at 4 mg/kg and VB12 at 1.5 mg/kg/day are supported by prior experimental or clinical dosing experience, no pilot dose-finding experiment or multi-dose comparison was performed in this study. Therefore, the current data cannot determine whether these doses represent the optimal dose combination, whether lower doses would be sufficient, or whether higher doses would provide additional benefit or toxicity. In addition, no formal a priori sample size calculation was performed. The group size of six rats per group was determined based on previous experience with similar LPS-induced septic shock models, experimental feasibility, the expected mortality of this acute model, and the ethical principle of reducing animal use. Thus, the study may be underpowered for some secondary endpoints, and the findings should be interpreted as preliminary experimental evidence. Moreover, all modeled animals received fluid resuscitation and norepinephrine after shock induction. Although this design improved model stability and maintained partial consistency with supportive care in septic shock, these interventions may also have influenced hemodynamic readouts. Therefore, the present results should be regarded as evidence from a controlled experimental model rather than direct proof of clinical efficacy.

In addition to parenchymal organ injury, the carotid artery histology provides evidence of vascular involvement in this model. This finding is pathophysiologically relevant because endothelial dysfunction is a central event in sepsis and septic shock. Sepsis can impair multiple endothelial functions, including vascular tone regulation, barrier integrity, inflammatory activation, and hemostatic balance [19]. In particular, degradation of the endothelial glycocalyx promotes vascular hyperpermeability, tissue edema, leukocyte adhesion, platelet aggregation, and dysregulated vasodilation [20]. The loss of endothelial and glycocalyx integrity also facilitates the transvascular leakage of albumin and fluids, contributing to vascular leak and impaired tissue perfusion [21]. In the present study, carotid artery alterations were accompanied by increased circulating Syndecan-1 and reduced serum albumin, which may reflect endothelial glycocalyx shedding and barrier dysfunction after LPS challenge. Together with the histological findings, the reductions in circulating Syndecan-1 and albumin loss after VB12 and/or MB administration may indicate attenuation of endothelial glycocalyx injury and barrier dysfunction. In parallel, the decreases in NO, H2S, and lactate may reflect reduced vasodilatory mediator burden and metabolic stress during LPS-induced septic shock. However, these interpretations remain associative and require further mechanistic validation.

Mechanistically, VB12 and MB may act at different levels of the NO-related gasotransmitter and sGC-cGMP-PRKG signaling cascade. During septic shock, inflammatory activation can increase NO generation, particularly through inducible nitric oxide synthase. Excessive NO then activates soluble guanylate cyclase in vascular smooth muscle cells, leading to increased cGMP production, PKG/PRKG activation, smooth muscle relaxation, and reduced vascular resistance [22,23]. MB counteracts excessive NO-mediated vasodilatory signaling, thereby attenuating pathological vasodilation; rather, one of its best-characterized vasoactive mechanisms is inhibition of NO-sensitive soluble guanylate cyclase, thereby limiting downstream cGMP accumulation and cGMP-dependent signaling [24]. In contrast, hydroxocobalamin/VB12 may reduce the bioavailability of vasodilatory gasotransmitters by directly interacting with or scavenging NO and by binding H2S/sulfide [25]. Therefore, the combined regimen may interrupt this pathway at complementary levels: VB12 may reduce upstream NO/H2S bioavailability, whereas MB may restrict downstream NO-stimulated sGC-cGMP-PRKG signaling. This two-level interruption provides a biologically plausible explanation for the observed decreases in NO, H2S, cGMP, PRKG1, and PRKG2, together with partial improvement in PVR and blood pressure parameters. Nevertheless, the present study does not prove pharmacological synergy between VB12 and MB, because formal interaction analysis, dose-response experiments, and direct target-engagement assays were not performed.

KCNMB1 may represent another vascular response-related marker in this model. KCNMB1 encodes the β1 regulatory subunit of large-conductance calcium-activated potassium channels and has been implicated in vascular smooth muscle function, vascular compliance, and blood pressure regulation [26]. Previous studies have shown that KCNMB1 is related to pulmonary vascular smooth muscle function. Under hypoxic conditions, HIF-1α binds to hypoxia-response elements within the KCNMB1 promoter and upregulates its expression, which may help counteract cytosolic calcium overload and vasoconstriction [27]. In septic shock, tissue oxygen supply-demand imbalance, microcirculatory dysfunction, mitochondrial impairment, and inflammatory vascular injury may all contribute to vascular stress responses. In this context, KCNMB1 upregulation in the model group may reflect a vascular adaptive or stress-related response rather than a direct pathogenic driver. PRKG1 and PRKG2 encode cyclic GMP-dependent protein kinases, specifically cGKI and cGKII, which are major downstream effectors of the NO/cGMP signaling pathway [28]. cGKI is closely involved in smooth muscle relaxation, whereas cGKII has been associated with regulation of renin secretion. The observed reductions in KCNMB1, PRKG1, and PRKG2 after combined VB12 and MB treatment may therefore reflect attenuation of vascular stress and cGMP-dependent signaling activity. However, whether KCNMB1 functionally interacts with the NO/sGC/cGMP/PRKG axis in septic shock remains to be determined.

In summary, combined MB and VB12 pretreatment attenuated hemodynamic disturbance, tissue injury, systemic inflammation, oxidative stress, and vascular injury-related changes in a rat model of LPS-induced septic shock. These effects were accompanied by reduced NO, H2S, cGMP, KCNMB1, PRKG1, and PRKG2 levels, supporting the hypothesis that the combined regimen may interfere with both upstream vasodilatory gasotransmitter bioavailability and downstream sGC-cGMP-PRKG signaling. However, the present study does not establish a direct causal link between these molecular changes and the observed physiological benefits. Future studies should include post-insult treatment designs, dose-response analyses, NOS isoform detection, sGC subunit assessment, phosphodiesterase activity measurement, endothelial marker staining, and pathway-intervention experiments to further validate this proposed mechanism.

## Supporting information

S1 Data Numerical data underlying the figures. (XLSX)

S1 File Original uncropped and unadjusted western blot images underlying Fig 2B. (PDF)

## Abbreviations

MB: methylene blue
VB12: vitamin B12
NO: nitric oxide
sGC: soluble guanylate cyclase
cGMP: cyclic guanosine monophosphate
H_2_S: hydrogen sulfide
LPS: lipopolysaccharide
MAP: mean arterial pressure
CO: cardiac output
TMB: 3,3’,5,5’-tetramethylbenzidine
IHC: immunohistochemistry
PCA: principal component analysis
DEGs: differentially expressed genes
KCNMB1: potassium calcium-activated channel subfamily M regulatory beta subunit 1
PRKG1/2: protein kinase cGMP-dependent 1/2
cGKs: cyclic GMP-dependent protein kinases
cGKI: cyclic GMP-dependent protein kinase I
cGKII: cyclic GMP-dependent protein kinase II
PDGFB: platelet-derived growth factor subunit B
TGFBR1: transforming growth factor beta receptor 1
ALB: albumin
SBP: systolic blood pressure
DBP: diastolic blood pressure
PVR: peripheral vascular resistance
LA: lactate
SOD: superoxide dismutase
GSH-Px: glutathione peroxidase
CAT: catalase
HIF-1α: hypoxia-inducible factor 1-alpha

## References

[1] Prescott HC, Antonelli M, Alhazzani W, Møller MH, Alshamsi F, Azevedo LCP, et al. Surviving Sepsis Campaign: International Guidelines for Management of Sepsis and Septic Shock 2026. Critical Care Medicine. 2026 [cited 16 June 2026]. doi: 10.1097/CCM.0000000000007075 41869847

[2] De Backer D, Ricottilli F, Ospina-Tascón GA. Septic shock: a microcirculation disease. Curr Opin Anaesthesiol. 2021;34(2):85–91. doi: 10.1097/ACO.0000000000000957 33577205

[3] Meyer NJ, Prescott HC. Sepsis and septic shock. Hardin CC, editor. N Engl J Med. 2024;391: 2133–46. doi: 10.1056/NEJMra2403213 39774315

[4] Fernando SM, Tran A, Soliman K, Flynn B, Oommen T, Wenzhe L, et al. Methylene blue in septic shock: a systematic review and meta-analysis. Crit Care Explor. 2024;6(7):e1110. doi: 10.1097/CCE.0000000000001110 38904978 PMC11196076

[5] Heemskerk S, van Haren FMP, Foudraine NA, Peters WHM, van der Hoeven JG, Russel FGM, et al. Short-term beneficial effects of methylene blue on kidney damage in septic shock patients. Intensive Care Med. 2008;34(2):350–4. doi: 10.1007/s00134-007-0867-9 17926021 PMC2228379

[6] Saha BK, Burns SL. The story of nitric oxide, sepsis and methylene blue: a comprehensive pathophysiologic review. Am J Med Sci. 2020;360(4):329–37. doi: 10.1016/j.amjms.2020.06.007 32631574

[7] Kuzenkov VS, Krushinskii AL. Effect of Sodium Nitrite and L-NNA on the Outcome of Experimental Ischemic Stroke. Bull Exp Biol Med. 2015;159(2):217–20. doi: 10.1007/s10517-015-2926-5 26085355

[8] Li T, Bao B, Hao Y, Liu J, Bi H, Guo D. Suppressive effect of nitric oxide synthase (NOS) inhibitor L-NMMA acetate on choroidal fibrosis in experimental myopic guinea pigs through the nitric oxide signaling pathway. Eur J Pharmacol. 2023;960:176111. doi: 10.1016/j.ejphar.2023.176111 37863413

[9] Ballarin RS, Lazzarin T, Zornoff L, Azevedo PS, Pereira FWL, Tanni SE, et al. Methylene blue in sepsis and septic shock: a systematic review and meta-analysis. Front Med (Lausanne). 2024;11:1366062. doi: 10.3389/fmed.2024.1366062 38698779 PMC11063345

[10] Hernández Díaz ZA, Hernández Puentes JS, Bilbao Rubiano LD, Granados Burgos CA, Caycedo VP, Bastidas Goyes AR. Use of methylene blue in early septic shock: A scoping review. Journal of Emergency Medicine, Trauma & Acute Care. 2026; 2026: 14. doi: 10.5339/jemtac.2026.14

[11] Zhao C-C, Zhai Y-J, Hu Z-J, Huo Y, Li Z-Q, Zhu G-J. Efficacy and safety of methylene blue in patients with vasodilatory shock: A systematic review and meta-analysis. Front Med (Lausanne). 2022;9:950596. doi: 10.3389/fmed.2022.950596 36237547 PMC9552293

[12] Fissoun C, Kovatcheva M. Vitamin B12 in Cell Plasticity and Repair. DNA Cell Biol. 2025;44(5):209–13. doi: 10.1089/dna.2025.0037 40106269

[13] Shapeton AD, Mahmood F, Ortoleva JP. Hydroxocobalamin for the Treatment of Vasoplegia: A Review of Current Literature and Considerations for Use. J Cardiothorac Vasc Anesth. 2019;33(4):894–901. doi: 10.1053/j.jvca.2018.08.017 30217583

[14] Patel JJ, Willoughby R, Peterson J, Carver T, Zelten J, Markiewicz A, et al. High-Dose IV Hydroxocobalamin (Vitamin B12) in Septic Shock. CHEST. 2023;163(2):303–12. doi: 10.1016/j.chest.2022.09.021 36174744

[15] Shaker EH, Soliman AM, Bedewy AAE, Elrawas MM. Comparative study between high and low dose methylene blue infusion in septic cancer patients: a randomized, blinded, controlled study. BMC Anesthesiol. 2025;25(1):15. doi: 10.1186/s12871-024-02792-3 39780053 PMC11707904

[16] Mestriner F, Dantas PB, Michelon-Barbosa J, Dugaich VF, Luis-Silva F, Ribeiro MS, et al. Methylene blue as a potential intervention in sepsis: Effects on survival and microcirculation in rat models of sepsis. Biomed Pharmacother. 2025;187:118131. doi: 10.1016/j.biopha.2025.118131 40349555

[17] Abdulwahhab RQ, Ali Alabdali SM. Study of the protective effects of cyanocobalamin on methotrexate induced nephrotoxicity in rats. F1000Res. 2022;11: 1012. doi: 10.12688/f1000research.124081.2 36405556 PMC9641110

[18] Foster DM, Kellum JA. Endotoxic Septic Shock: Diagnosis and Treatment. Int J Mol Sci. 2023;24(22):16185. doi: 10.3390/ijms242216185 38003374 PMC10671446

[19] Ince C, Mayeux PR, Nguyen T, Gomez H, Kellum JA, Ospina-Tascón GA, et al. The Endothelium in Sepsis. Shock. 2016;45: 259–70. doi: 10.1097/SHK.0000000000000473 26871664 PMC5281063

[20] Uchimido R, Schmidt EP, Shapiro NI. The glycocalyx: a novel diagnostic and therapeutic target in sepsis. Crit Care. 2019;23(1):16. doi: 10.1186/s13054-018-2292-6 30654825 PMC6337861

[21] McMullan RR, McAuley DF, O’Kane CM, Silversides JA. Vascular leak in sepsis: physiological basis and potential therapeutic advances. Crit Care. 2024;28(1):97. doi: 10.1186/s13054-024-04875-6 38521954 PMC10961003

[22] Aronsson P, Stenqvist J, Ferizovic E, Danielsson E, Jensen A, Simonsen U, et al. Soluble guanylate cyclase mediates the relaxation of healthy and inflamed bladder smooth muscle by aqueous nitric oxide. Front Physiol. 2023;14:1249560. doi: 10.3389/fphys.2023.1249560 37731544 PMC10507315

[23] Lincoln TM, Dey N, Sellak H. Invited review: cGMP-dependent protein kinase signaling mechanisms in smooth muscle: from the regulation of tone to gene expression. J Appl Physiol (1985). 2001;91(3):1421–30. doi: 10.1152/jappl.2001.91.3.1421 11509544

[24] Arias-Ortiz J, Vincent J-L. Administration of methylene blue in septic shock: pros and cons. Crit Care. 2024;28(1):46. doi: 10.1186/s13054-024-04839-w 38365828 PMC10870439

[25] Patel JJ, Venegas-Borsellino C, Willoughby R, Freed JK. High-Dose Vitamin B12 in Vasodilatory Shock: A Narrative Review. Nutr Clin Pract. 2019;34(4):514–20. doi: 10.1002/ncp.10327 31187494

[26] Son Y, Chun W, Ahn Y-T, Kim K, Lee C-W, Kim J-M, et al. 7-Ketocholesterol induces the reduction of KCNMB1 in atherosclerotic blood vessels. Biochem Biophys Res Commun. 2015;457(3):324–7. doi: 10.1016/j.bbrc.2014.12.109 25576871

[27] Ahn Y-T, Kim Y-M, Adams E, Lyu S-C, Alvira CM, Cornfield DN. Hypoxia-inducible factor-1α regulates KCNMB1 expression in human pulmonary artery smooth muscle cells. Am J Physiol Lung Cell Mol Physiol. 2012;302(3):L352-9. doi: 10.1152/ajplung.00302.2011 22114151 PMC3289270

[28] Hofmann F, Wegener JW. cGMP-dependent protein kinases (cGK). Methods Mol Biol. 2013;1020:17–50. doi: 10.1007/978-1-62703-459-3_2 23709024

