# Supplementary material for: Vitamin B12 combined with methylene blue alleviates experimental septic shock and is associated with modulation of KCNMB1 and the cGMP-PRKG pathway
Source: PLoS One. 2026 Aug 6;21(8):e0355117. doi: 10.1371/journal.pone.0355117 (PMC13446705; doi:10.1371/journal.pone.0355117)
Supplement: Supplementary file 1 [file pone.0355117.s002.pdf]

Figure2B

KCNMB

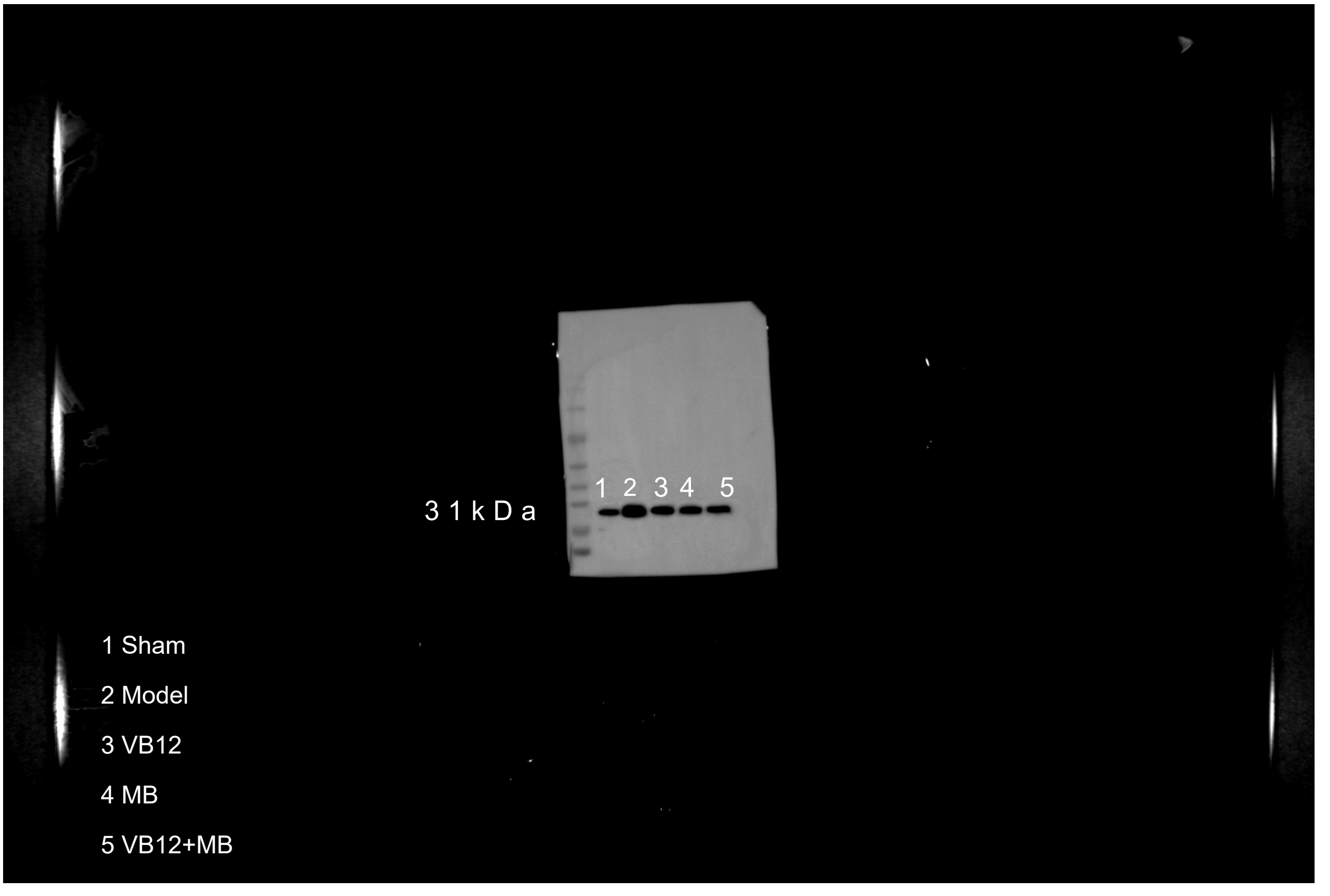

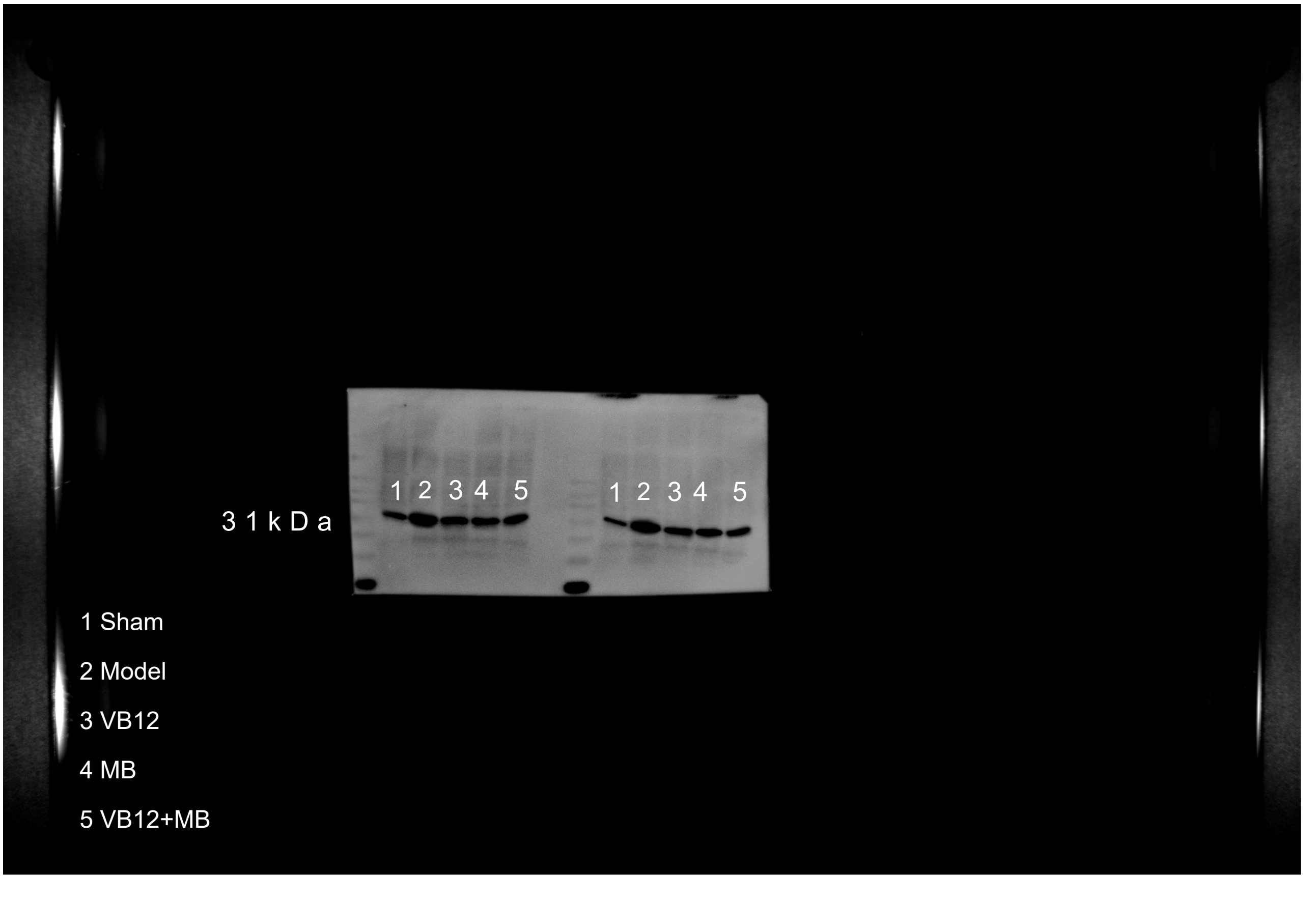

PRKG2

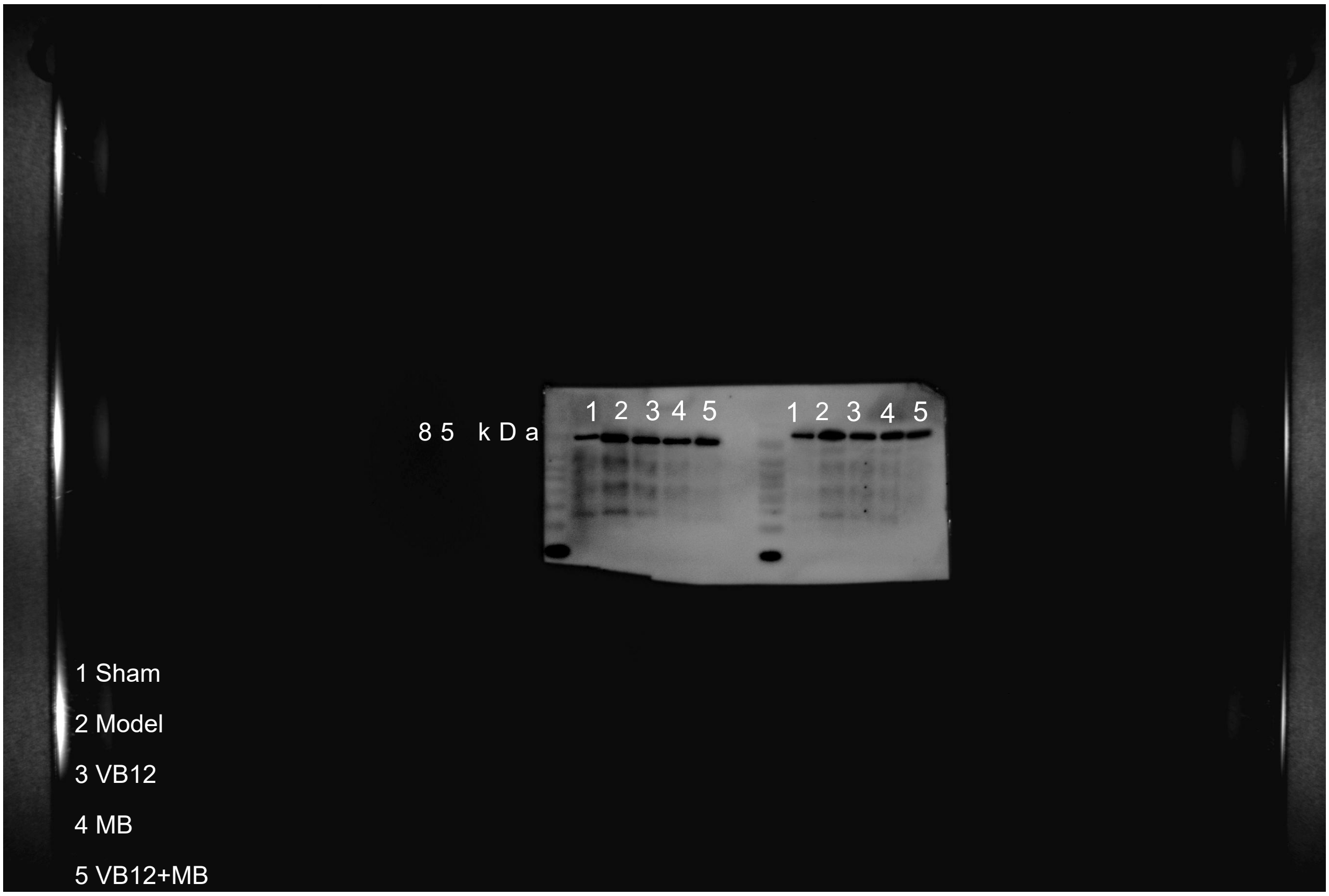
85 kDa
1 2 3 4 5
1 2 3 4 5
1 Sham
2 Model
3 VB12
4 MB
5 VB12+MB

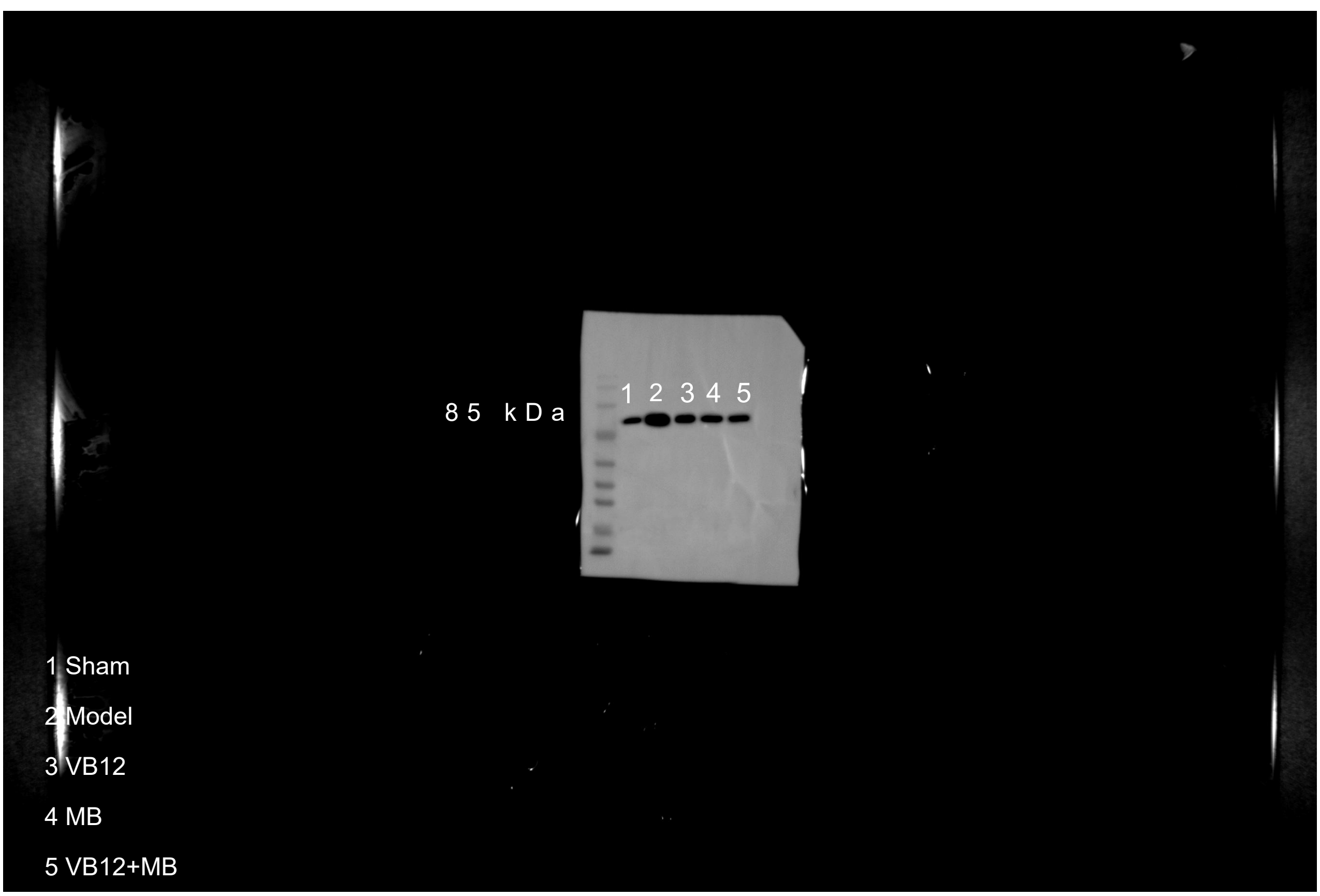
85 kDa
1 2 3 4 5
1 Sham
2 Model
3 VB12
4 MB
5 VB12+MB

PRKG1

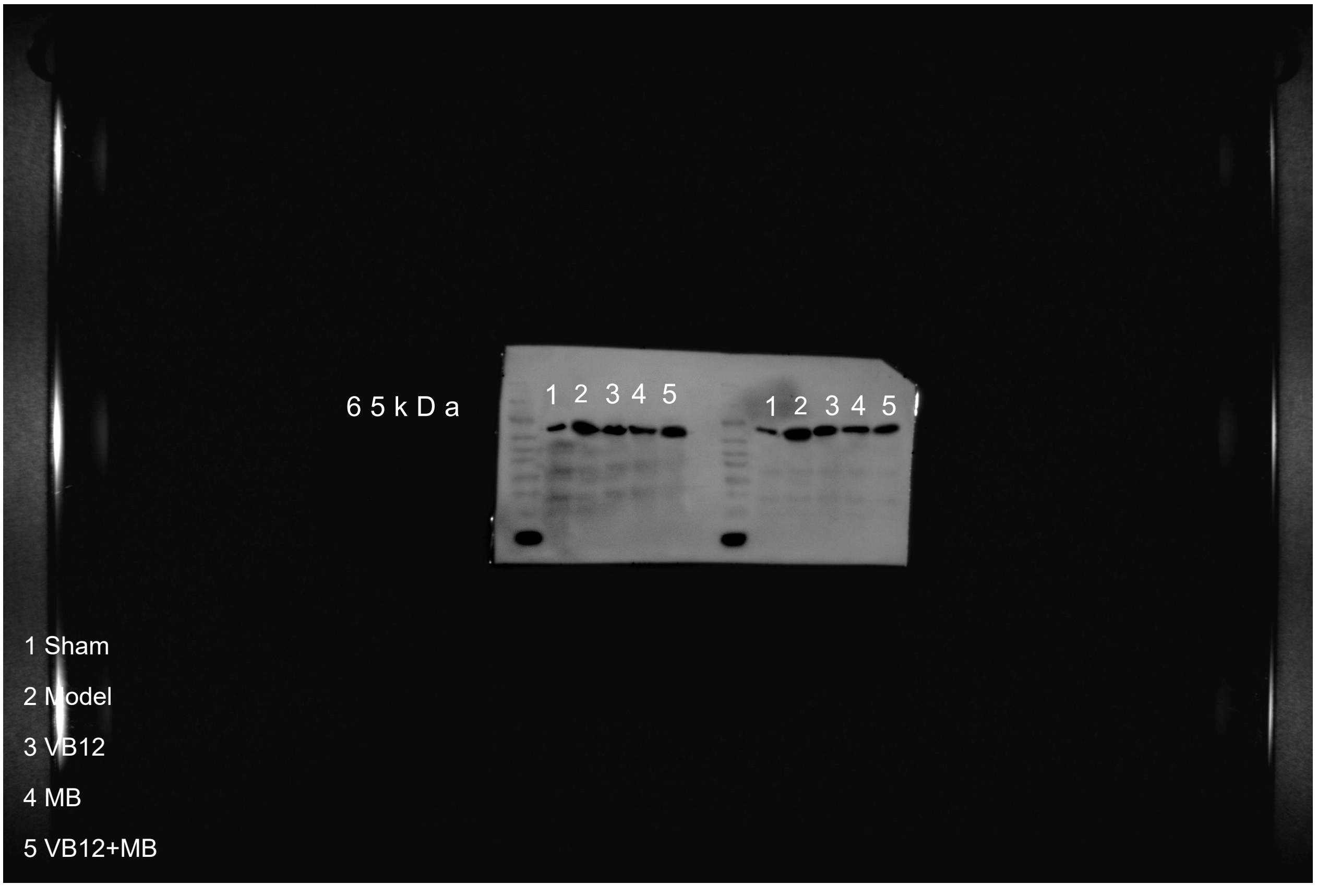
65kDa
1 2 3 4 5
1 2 3 4 5
1 Sham
2 Model
3 VB12
4 MB
5 VB12+MB

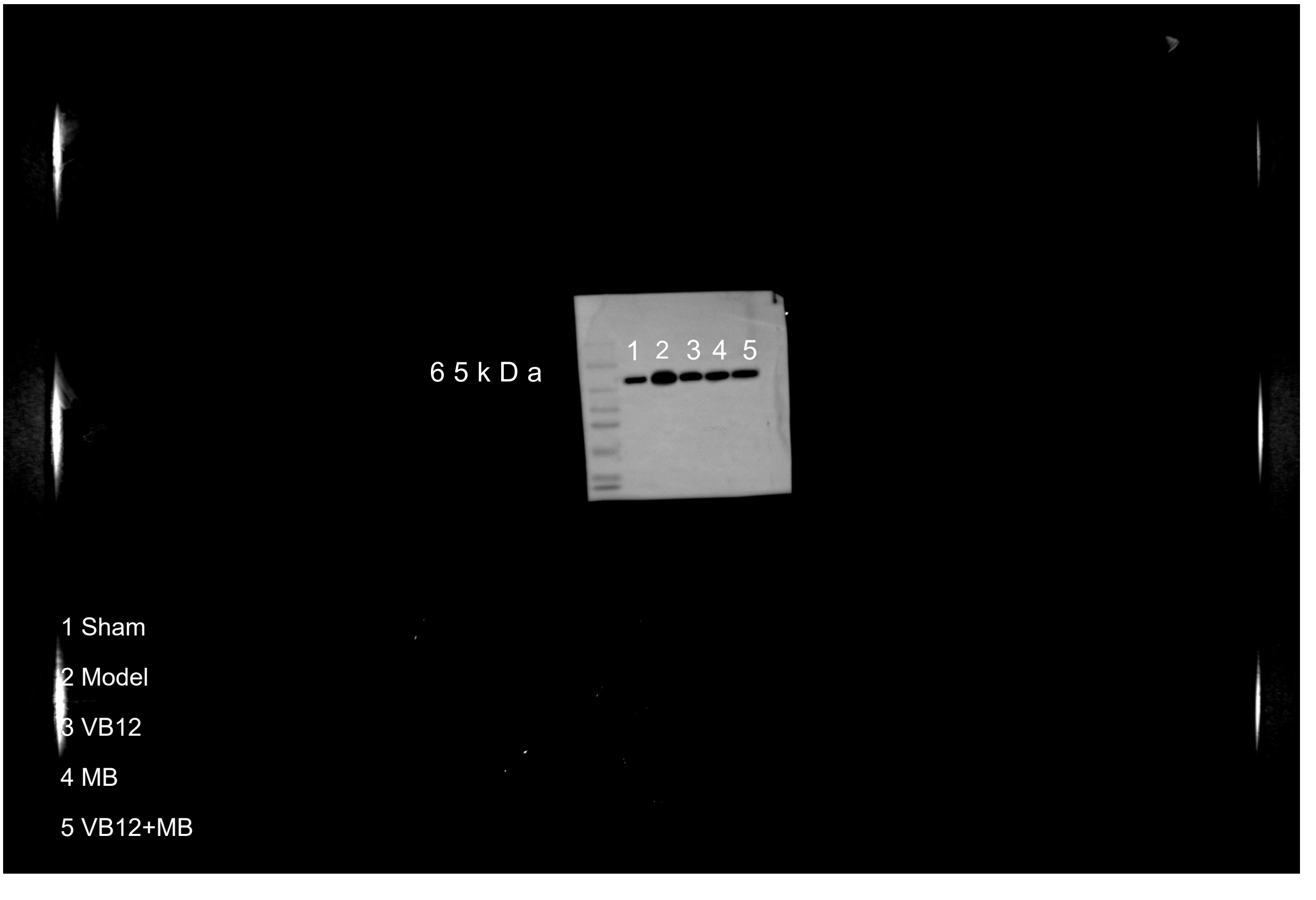
65kDa
1 2 3 4 5
1 Sham
2 Model
3 VB12
4 MB
5 VB12+MB

β-actin

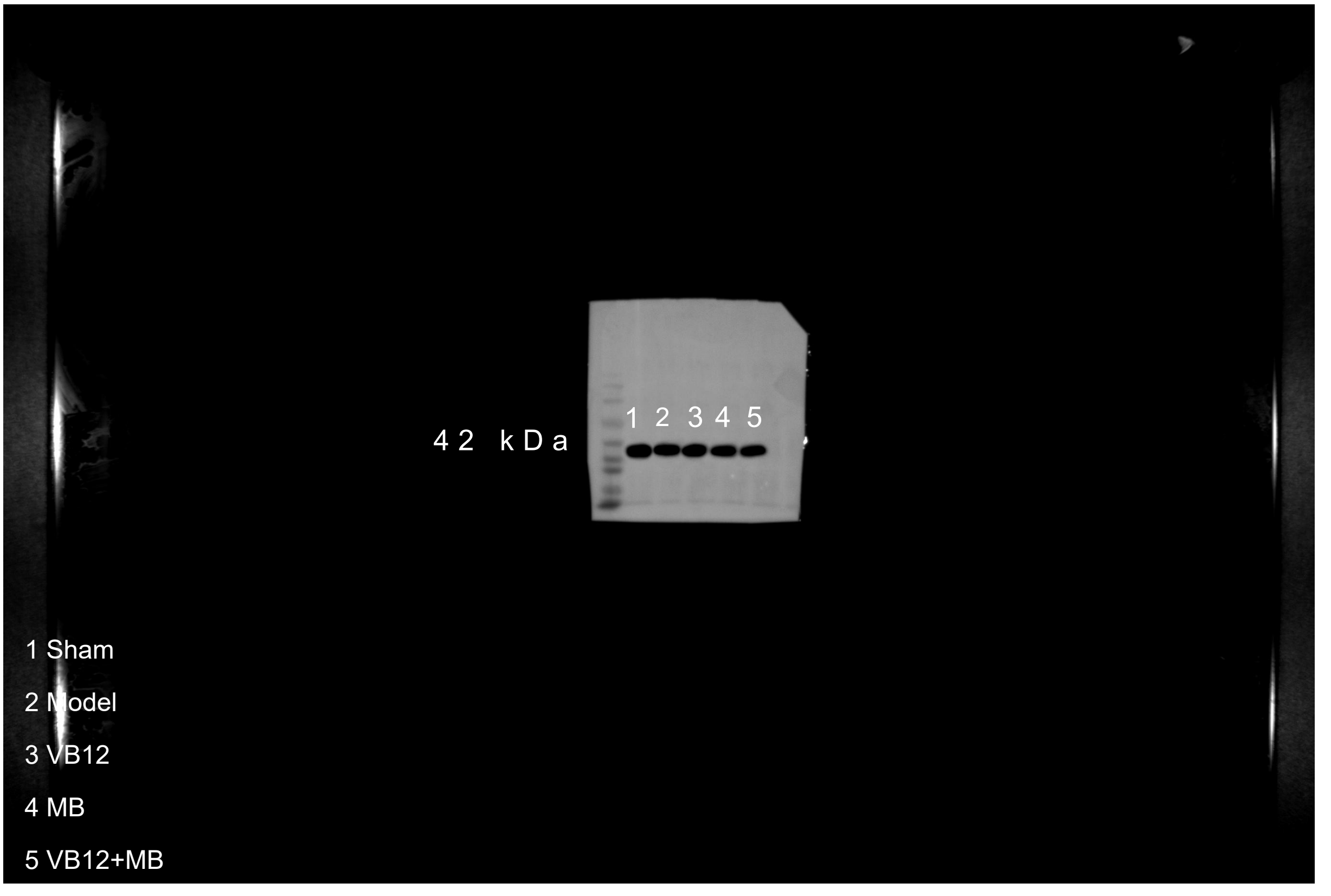
42 kDa
1 2 3 4 5
1 Sham
2 Model
3 VB12
4 MB
5 VB12+MB

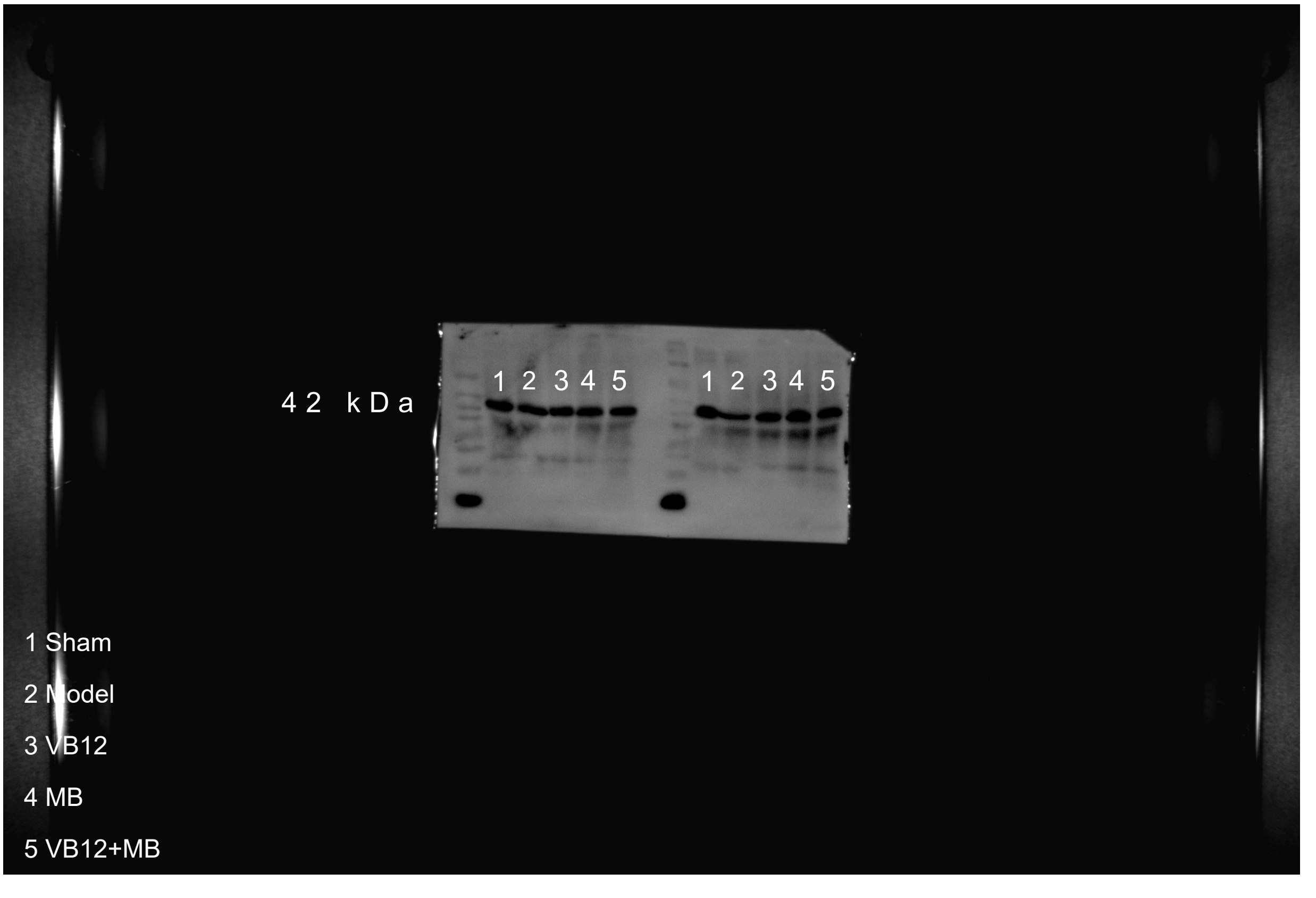
42 kDa
1 2 3 4 5
1 2 3 4 5
1 Sham
2 Model
3 VB12
4 MB
5 VB12+MB
